# Adherence to oral antineoplastic therapy among patients with advanced or metastatic non-small cell lung cancer: a noninterventional, prospective study

**DOI:** 10.1007/s00432-025-06264-0

**Published:** 2025-08-01

**Authors:** Irene Mangues-Bafalluy, Beatriz Bernardez, José Manuel Martínez-Sesmero, Andres Navarro-Ruiz, Maria Teresa Martín-Conde, Ana Rosa Rubio-Salvador, Judith Rius-Perera, Marta Gilabert-Sotoca, Marta Domínguez López, Angel Callejo Mellén

**Affiliations:** 1 Servicio de Farmacia, Hospital Universitari Arnau de Vilanova Lleida, Lleida, Spain; 2 Servicio de Farmacia, Complejo Hospitalario Universitario de Santiago de Compostela, Santiago de Compostela, Spain; 3 Servicio de Farmacia, Hospital Clínico Universitario Lozano Blesa, Saragossa, Spain; 4 Servicio de Farmacia, Hospital General Universitario de Elche, Alicante, Spain; 5 Servicio de Farmacia, Hospital Clínic de Barcelona, Barcelona, Spain; 6 Servicio de Farmacia, Complejo Hospitalario de Toledo, Toledo, Spain; 7 AstraZeneca Farmacéutica Spain, Madrid, Spain

**Keywords:** Non-small cell lung cancer, Oral chemotherapy, Tyrosine kinase inhibitors, Anaplastic lymphoma kinase inhibitors, Adherence, Progression-free survival

## Abstract

**Purpose:**

We aimed to evaluate adherence to oral antineoplastic therapy (OAT) in patients with locally advanced or metastatic non-small cell lung cancer (NSCLC) and its potential relationship with several clinical outcomes.

**Methods:**

Observational, prospective, multicenter study performed by 6 hospital pharmacists in Spain. The primary outcome was the proportion of treatment adherence as evaluated by pill reconciliation during the 3-month active follow-up period. Those with an adherence > 80% were categorized as adherent. We performed multivariate Cox regression analyses to explore the factors associated with progression-free survival.

**Results:**

From December 2019 to November 2022, we recruited 95 evaluable patients. Most of the patients received osimertinib (n = 45, 45.3%) or a first- or second-generation tyrosine kinase inhibitor (n = 23, 34.8%). Eighty-one patients showed greater than 80% adherence (85.3%, 95% CI 78.1% to 92.4%), as evaluated based on pill reconciliation; the mean (SD) adherence to OAT was 94.7% (11.4). According to the univariate analysis, the time to progression from study entry was significantly shorter among patients who were nonadherent than among those who were adherent (median 6.5 months vs. not reached, log-rank test *p* = 0.006; hazard ratio [HR] 2.619, 95% confidence interval [CI] 1.240–5.532). In the multivariate Cox regression analysis, nonadherence was the single factor associated with progression-free survival.

**Conclusion:**

Consistent with previous evidence in this setting, our results suggest that adherence to oral antineoplastic treatment among patients with NSCLC is high. Whether this high rate of adherence translates to better clinical outcomes should be further evaluated in larger samples.

**Supplementary Information:**

The online version contains supplementary material available at 10.1007/s00432-025-06264-0.

## Introduction

The availability and use of oral antineoplastic therapy (OAT) are increasing, accounting for almost half of the new cancer therapies marketed in the US in 2021 (Moreira et al. 2022; IQVIA Institute 2023). Although OAT has several advantages in terms of convenience for patients and is preferred over intravenous therapy (Weingart et al. 2008; Eek et al. 2016), it also poses some threats, such as the risk of medication errors, monitoring toxicity, and medication adherence (Weingart et al. 2008, 2010; Simchowitz et al. 2010).

A systematic review suggested that at least half of the patients diagnosed with cancer who are prescribed OAT are non-adherent (Greer et al. 2016). Although evidence is limited, poor adherence seems to be associated with poorer outcomes (McGrady and Pai 2019; Lasala and Santoleri 2022) and increased health care costs (Cutler et al. 2018). Despite its increasing role in the management of non-small cell lung cancer (NSCLC), adherence to OAT in this setting has scarcely been investigated. Most studies on adherence to OAT have been conducted in patients with breast cancer or hematological malignancies (Greer et al. 2016). Recently, some studies have reported adherence to OAT in patients with NSCLC but have focused on specific targeted therapies, such as tyrosine kinase inhibitors (TKIs) (Timmers et al. 2015; Hess et al. 2017; Rosentreter et al. 2021; Joret et al. 2022) or anaplastic lymphoma kinase inhibitors (ALKis) (Ganti et al. 2022). These studies reported high rates of adherence based on several outcome measures and explored some barriers and facilitators, but none explored the potential associations between adherence and clinical outcomes.

This study aimed to evaluate adherence to OAT in patients with locally advanced or metastatic NSCLC. We also assessed factors associated with adherence and the potential relationship between adherence and clinical outcomes.

## Materials and methods

### Study design and selection criteria

This was an observational, prospective, multicenter study performed by 6 hospital pharmacists in Spain. This study was approved by the Ethics Committee for Research with Medicines from Galicia (Santiago de Compostela, Spain), and all patients provided written informed consent.

Patients were included if they were 18 years or older, had been diagnosed with locally advanced or metastatic NSCLC, and were receiving or prescribed OAT for the management of NSCLC by the oncologist. Patients were excluded if, according to the investigator’s judgment, they were unable to understand the questionnaire administered during the study.

### Assessments

After inclusion, patients were actively followed-up over three months with four study visits, at baseline, and every month. Twelve months after patient inclusion, information on disease progression and OAT prescriptions and dispensations were recorded. At the time of inclusion, the pharmacist recorded information on demographics; disease-related variables, such as date of diagnosis; disease stage at the time of diagnosis; histology; ECOG performance status; number and location of metastases; biomarkers (specifically ALK, EGFR, EGFR-T790M, ROS-1, and BRAF); and treatment-related variables, including previous antineoplastic agents, concomitant medication, and OATs. At monthly visits, we recorded information on pill count, pharmaceutical interventions, and resource utilization, and patients completed the 4-item Morisky Green Levine Medication Adherence Scale (MGLS-4) (Jiménez et al. 1992). In addition, at the follow-up visits, patients completed other patient-reported outcomes (PROs), including the Brief Illness Perception Questionnaire (BIPQ) (Pacheco-Huergo et al. 2012) and the 3-level version of the EQ-5D (EQ-5D-3L) (Badia et al. 1999), which were administered at baseline and month 3; the results of these and other PROs will be published elsewhere.

The MGLS-4 is a brief self-administered questionnaire widely used to evaluate adherence in patients with chronic health conditions; it includes 4 yes/no questions on treatment adherence related to forgetfulness or the disease course (Jiménez et al. 1992). Each question is rated as 0 (no) or 1 (yes), and a score equal to or greater than 1 implies some degree of nonadherence.

### Statistical analysis

The sample size was based on the estimation of a proportion using the maximum variability criterion (i.e., an estimated prevalence of 50%) and a precision of 10%. Assuming a 10% loss, the sample size was estimated to be 106.

The primary outcome was the proportion of treatment adherence as evaluated by pill reconciliation during the 3-month active follow-up period. This parameter was calculated as the difference between the number of pills dispensed and the number of pills returned in the numerator, divided by the product of the number of days of treatment by the number of pills/day prescribed by the oncologist in the denominator, and multiplied by 100 to obtain the percentage of adherence. Patients with a percentage adherence > 80% were categorized as adherent and are referred to hereafter as such. Adherence based on a cutoff value of 90% was also calculated.

Adherence was also estimated via the proportion of days covered (PDC) and the MGLS-4. The PDC was calculated as the sum of days covered by the drug based on the prescription fill date and days of supply divided by the number of days of the treatment period (counted as the number of days between the index prescription dates until the end of the year of the study, study discontinuation, or death) and was multiplied by 100. Patients with a PDC ≥ 80% were considered adherent. Using the MGLS-4, patients were considered adherent if their score was 0.

Adherence as a continuous outcome is presented as the mean and standard deviation and as a binary outcome with absolute and relative frequencies and the corresponding 95% confidence interval.

We analyzed adherence to the following prespecified treatment subgroups: first- and second-generation EGFR-TKIs (erlotinib, afatinib and gefitinib), third-generation EGFR-TKIs (osimertinib), and ALK inhibitors (alectinib, crizotinib and lorlatinib).

The factors potentially associated with treatment adherence were explored via multiple logistic regression analysis, where the dependent variable was treatment adherence, as defined for the primary outcome, and the independent variables were as follows: age (continuous), sex, the presence of a person who reminds the patient daily or weekly to take the medication (binary), the anxiety‒depression item of the EQ-5D-3L (categorical), the ‘personal control’ item (“How much control do you feel you have over your illness?) of the BIPQ (continuous), the ‘treatment control’ item (‘How much do you think your treatment can help your illness?’) of the BIPQ (continuous), the ‘understanding’ item (‘How well do you feel you understand your illness?’) of the BIPQ (continuous), the number of concomitant medications (continuous), and the time since the first administration of oral antineoplastic treatment (continuous). This model was repeated excluding the time since the first administration of the oral antineoplastic treatment (months).

To explore the potential relationship between treatment adherence and progression-free survival, we used the Kaplan‒Meier method and the log-rank test to compare adherent and nonadherent patients based on pill count, and the effect size was calculated via univariate Cox regression analysis. Survival analysis was performed from the time of patient inclusion until 12 months after inclusion; as a sensitivity analysis, we also analyzed the time to progression from treatment initiation. In addition, we performed a post hoc multivariate Cox regression analysis to explore the factors associated with progression-free survival with the following independent variables: age (≤ 65/ > 65 years), sex (binary), Eastern Cooperative Oncology Group (ECOG-PS) score (categorical), tobacco consumption (categorical), presence of locally advanced/metastatic disease (binary), presence of central nervous system metastases (binary), molecular classification (binary: EGFR/ALK) and treatment adherence, as defined in the primary outcome (binary). As a sensitivity analysis, we also performed multivariate Cox regression analysis considering the time to progression from treatment initiation. A stepwise backward approach was used for fitting the regression models. All analyses were performed via IBM SPSS Statistics 26.0 and were considered significant if the *p* value was < 0.05.

## Results

**Fig. 1:**
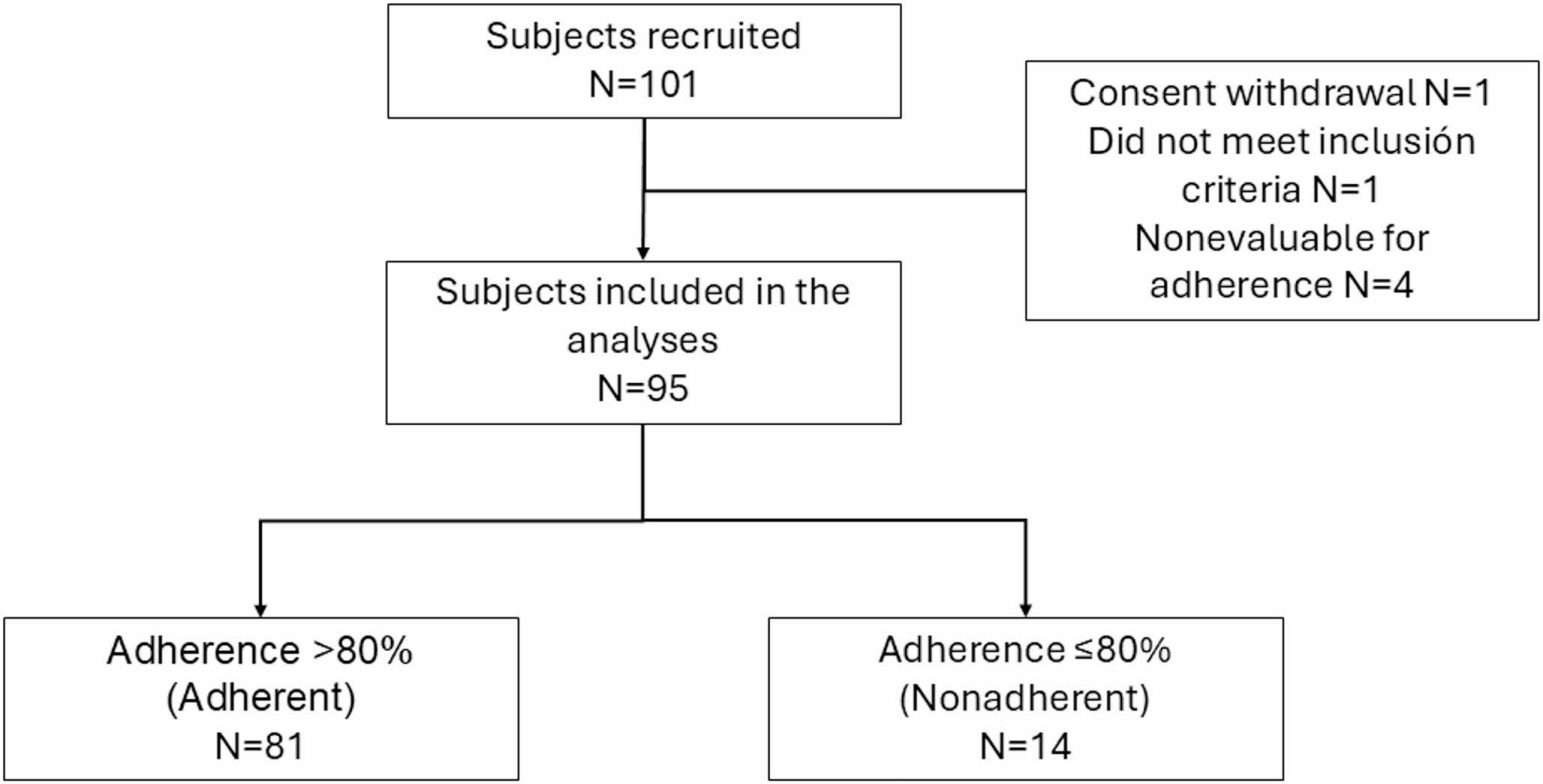
Patient disposition

From December 2019 to November 2022, we recruited 101 patients, of whom 2 were screening failures and 4 were not evaluable for adherence, leading to 95 evaluable patients (Fig. 1); unless otherwise specified, these evaluable patients were included in all analyses. The patients had a mean (SD) age of 66.3 (13.1) years and were predominantly women (n = 63, 66.3%) (Table 1). Over 90% of the patients had Stage IV disease and had an ECOG-PS of 0–1 at the time of study entry. Half of the patients had previously received therapy for NSCLC, and 83 (87.4%) had already received OAT at the time of inclusion. Among the 95 evaluable patients, 43 (45.3%) received the third-generation TKI osimertinib, 23 (34.8%) received a first- or second-generation TKI, 27 (28.4%) received an ALK inhibitor, and 2 (2.1%) were treated with nintedanib.

**Table 1:** Demographic and clinical characteristics

	All N = 95	Adherent N = 81	Nonadherent N = 14
**Age, mean (SD)**	66.3 (13.1)	67.2 (12.8)	61.6 (14.2)
**Sex (female), n (%)**	63 (66.3)	52 (64.2)	11 (78.6)
**Race (Caucasian), n (%)**	94 (98.9)	80 (98.8)	14 (100)
**Educational level**			
Primary education	45 (47.4)	36 (44.4)	9 (64.3)
Secondary education	15 (15.8)	13 (16.0)	2 (14.3)
Tertiary education	1 (1.1)	0 (0.0)	1 (7.1)
Professional training	12 (12.6)	11 (13.6)	1 (7.1)
University education	8 (8.4)	8 (9.9)	0 (0.0)
Uneducated	9 (9.5)	9 (11.1)	0 (0.0)
Unknown	5 (5.3)	4 (4.9)	1 (7.1)
**Tobacco consumption**			
No smoker	55 (57.9)	45 (55.6)	10 (71.4)
Former smoker	30 (31.6)	26 (32.1)	4 (28.6)
Smoker	10 (10.5)	10 (12.3)	0 (0.0)
**Time since diagnosis (months), mean (SD)**	31.9 (46.3)	30.0 (45.7)	43.0 (49.9)
**Current disease stage, n (%)**			
IIIA	2 (2.1)	1 (1.2)	1 (7.1)
IIIB	3 (3.2)	2 (2.5)	1 (7.1)
IIIC	3 (3.2)	3 (3.7)	0 (0.0)
IVA	52 (54.7)	42 (51.9)	10 (71.4)
IVB	35 (36.8)	33 (40.7)	2 (14.3)
**ECOG-PS, n (%)**			
0	49 (51.6)	45 (55.6)	4 (28.6)
1	40 (42.1)	33 (40.7)	7 (50.0)
2	5 (5.3)	2 (2.5)	3 (21.4)
3	1 (1.1)	1 (1.2)	0 (0.0)
**Metastases (yes), n (%)**	86 (90.5)	74 (91.4)	12 (85.7)
**Previous therapy, n (%)**	47 (49.5)	34 (42.0)	13 (92.9)
**Previous OAT (yes), n (%)**	83 (87.4)	72 (88.9)	11 (78.6)
**Time since first OAT (months)**^**†**^**, mean SD**	14.4 (19.2)	12.8 (16.7)	24.7 (30.1)
**Concomitant medication (yes),**	78 (82.1)	66 (81.5)	12 (85.7)
**Oral concomitant medication (yes), n (%)**	75 (78.9)	63 (77.8)	12 (85.7)
**Oral antineoplastic therapy**			
* 1st & 2nd Generation TKIs*	23 (34.8)	21 (35.0)	2 (14.2)
Afatinib	10 (10.5)	9 (11.1)	1 (7.1)
Erlotinib	5 (5.3)	5 (6.2)	0 (0.0)
Gefitinib	8 (8.4)	7 (8.6)	1 (7.1)
* 3er Generation TKIs*	43 (45.3)	39 (48.1)	4 (28.6)
Osimertinib	43 (45.3)	39 (48.1)	4 (28.6)
* ALK inhibitors*	27 (28.4)	20 (24.7)	7 (50.0)
Alectinib	17 (17.9)	15 (18.5)	2 (14.3)
Crizotinib	5 (5.3)	2 (2.5)	3 (21.4)
Lorlatinib	5 (5.3)	3 (3.7)	2 (14.3)
* Other*	2 (2.1)	1 (7.1)	1 (7.1)
Nintedanib	2 (2.1)	1 (1.2)	1 (7.1)

^†^Twelve patients did not undergo OAT at the time of inclusion, therefore the number of evaluable patients for this variable was n = 83 (adherent n = 72, nonadherent n = 11)

ALK, anaplastic lymphoma kinase; ECOG-PS, Eastern Cooperative Oncology Group Performance Status; OAT, oral antineoplastic therapy; SD, standard deviation; TKI, tyrosine kinase inhibitor

Eighty-one patients showed adherence greater than 80% (85.3%, 95% CI 78.1–92.4%) as evaluated with pill reconciliation; the mean (SD) adherence to OAT was 94.7% (11.4) (Table 2). The mean (SD) adherence to OAT among adherent and nonadherent patients was 98.5% (6.3) and 72.4% (8.3), respectively.

**Table 2:** Treatment adherence to oral antineoplastic treatment for the total sample and by treatment subgroup

	Total N = 95	1st– 2nd generation TKIs N = 23	3rd generation TKIs N = 43	ALKis N = 27
Primary outcome– Pill reconciliation (> 80%)^†^				
> 80%, n (%), [95% CI]	81 (85.3) [78.1; 92.4]	21 (91.3) [79.8; 100]	39 (90.7) [82; 99.4]	20 (74.1) [57.5; 90.6]
Mean (SD)	94.7 (11.4)	95.3 (11.6)	95.9 (8.9)	92.6 (14.5)
Secondary outcomes				
	Total N = 95	1st– 2nd generation TKIs N = 23	3rd generation TKIs N = 43	ALKis N = 27
*Pill reconciliation (*> *90%)* n (%) [95% CI]	76 (80.0) [72.0; 88.0]	20 (87.0) [73.2; 100]	37 (86.0) [75.7; 96.4]	18 (66.7) [48.9; 84.4]
*PDC* > *80%* N (%) [95% CI]	Total N = 99	1st– 2nd generation TKIs N = 24	3rd generation TKIs N = 44	ALKis N = 29
	86 (86.9) [80.2; 93.5]	21 (87.5) [74.3; 100]	39 (88.6) [79.3; 98]	25 (86.2) [73.7; 98.8]
*Morisky green* n (%) [95% CI]	Total N = 92	1st– 2nd generation TKIs N = 21	3rd generation TKIs N = 42	ALKis N = 27
	72 (78.3) [69.8; 86.7]	15 (71.4) [52.1; 90.8]	35 (83.3) [72.1; 94.6]	21 (77.8) [62.1; 93.5]

^†^Missing data for treatment subgroup analyses: n = 1 for each study subgroup

Demographic and clinical characteristics of adherent and nonadherent patients are described in Table 1. TKIs had similar adherence rates according to measures based on pill reconciliation and the PDC. ALKis had lower adherence rates according to the outcomes based on pill reconciliation, with 20 of 27 patients (74.1%, 95% CI 57.5–90.6%) showing an adherence > 80% but similar adherence rates to the other therapies based on the PDC (Table 2). Adherence rates based on the MGLS-4 were consistently lower and showed greater variability across treatment groups than with the other outcome measures for assessing adherence, with the proportion of adherent patients ranging from 71.4% for first- and second-generation TKIs to 83.3% for osimertinib (Table 2).

In the multivariate logistic regression analysis, when the time from treatment initiation of OAT was considered, the factors associated with treatment adherence greater than 80% after backward selection of variables were age (OR 1.039, 95% CI 1.024–1.054, *p* < 0.001) and the time from treatment initiation of OAT (OR 0.971, 95% CI 0.944–0.999, *p* = 0.044) (see Supplementary Table 1 for the initial model). When the analysis was performed without considering the time from the initiation of OAT, the factors associated with a treatment adherence greater than 80% after the backward selection of variables were age (OR 1.045, 95% CI 1.025–1.065, *p* < 0.001) and the number of concomitant medications (OR 0.837, 95% CI 0.714–0.981) (see Supplementary Table 2 for the initial model).

**Fig. 2:**
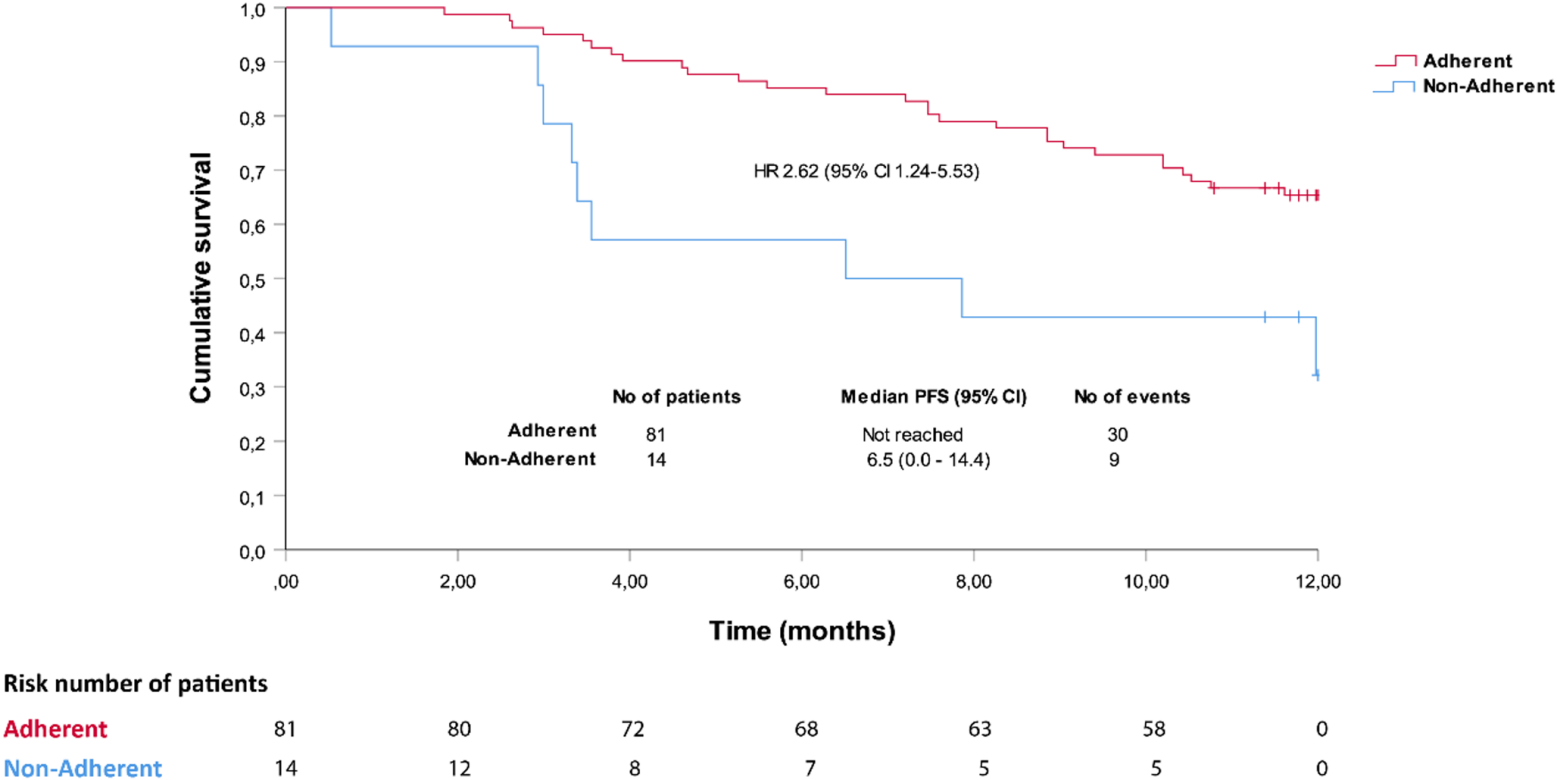
Time to progression in adherent and nonadherent patients receiving oral antineoplastic treatment for non-small cell lung cancer from their inclusion in the study. CI, confindence interval; HR, hazard ratio; PFS, progression-free survival

The time to progression from study entry was significantly shorter among patients who were nonadherent than among those who were adherent (median 6.5 months vs. not reached, log-rank test *p* = 0.006; HR 2.619, 95% CI 1.240–5.532) (Fig. 2). In the multivariate Cox regression analysis, nonadherence was the single factor associated with progression-free survival (PFS) in the initial model (HR 3.043, 95% CI 1.126–8.218) (Table 3); after backward selection of variables, nonadherence was the single variable that was maintained in the model (HR 2.619, 95% CI 1.240–5.532). When analyzed from treatment initiation, the time to progression among those who were nonadherent was also shorter than that among those who were adherent, but the differences were not statistically significant (median 18.2 vs. 45.6 months, log-rank test *p* = 0.145: HR 1.609, 95% CI 0.745–3.474) (Supplementary Fig. 1). Using this second approach (i.e., considering time to progression from treatment initiation), none of the factors were significantly associated with PFS in the multivariate Cox regression analysis, neither in the initial model nor after backward selection of variables (data not shown).

**Table 3:** Multiple Cox regression analysis of the factors associated with progression-free survival (initial model).

Variable	β	SE	Wald	df	*p* value	HR	95% CI
Age, ≤ 65 years (> 65 years)^†^	0.062	0.380	0.027	1	0.869	1.064	0.506–2.241
Sex (male)^†^	−0.042	0.421	0.010	1	0.921	0.959	0.420–2.191
ECOG-PS (ECOG-PS 2–3)^†^			0.122	2	0.941		
ECOG-PS (0)	−0.197	0.936	0.044	1	0.833	0.821	0.131–5.143
ECOG-PS (1)	−0.070	0.909	0.006	1	0.939	0.933	0.157–5.534
Tobacco consumption (Smoker)^†^			0.182	2	0.913		
Tobacco consumption (Former smoker)	−0.188	0.730	0.067	1	0.796	0.828	0.198–3.463
Tobacco consumption (No smoker)	−0.020	0.684	0.001	1	0.977	0.980	0.256–3.748
Locally advanced/metastatic (Yes)^†^	−1.133	0.880	1.659	1	0.198	0.322	0.057–1.806
CNS metastasis (Yes)^†^	0.170	0.492	0.119	1	0.730	1.185	0.452–3.108
Molecular classification, ALK (EGFR)^†^	−0.488	0.492	0.984	1	0.321	0.614	0.234–1.610
Adherence 80% (yes)^†^	1.113	0.507	4.818	1	0.028	3.043	1.126–8.218

^†^Reference categories appear in brackets

CI, confidence interval; CNS, central nervous system; df, degrees of freedom; ECOG-PS, Eastern Cooperative Oncology Group performance status; SE, standard error

Eight (9.9%) of the 81 patients who were adherent required hospital admission during the study period, whereas 2 (14.3%) of the 14 patients who were not adherent (*p* = 0.779) did. Seven (8.6%) of the 81 patients who were adherent needed to visit the emergency department compared to 1 (7.1%) of 14 patients who were not adherent (comparison could not be made).

## Discussion

Our results show that adherence to OAT in patients with NSCLC is high regardless of the type of treatment. Adherence to OAT seems to be associated with a reduced likelihood of progression without affecting hospitalizations or emergency visits.

Previous studies have shown great variability in adherence to OAT. A previous systematic review of adherence to OAT that included 51 studies, mostly conducted in patients with breast cancer or hematologic malignancies, revealed that the proportion of adequate adherence ranged from 45 to 100%, depending on several factors, including the type of sample, medication type, duration of follow-up, and method for assessment of adherence (Greer et al. 2016). However, this systematic review did not include studies of patients with NSCLC (Greer et al. 2016). Our results are consistent with those of recent studies on adherence in patients with NSCLC treated with TKIs or ALKis (Timmers et al. 2015; Hess et al. 2017; Ganti et al. 2022). Thus, the proportion of patients who are adherent to erlotinib has been reported to be 87% in a retrospective study using a medication possession ratio (MPR) ≥ 80% (Hess et al. 2017) and 92.7% in a prospective study using a medication event monitoring system, with adherence defined as a PDC ≥ 90% (Timmers et al. 2015). A study evaluating adherence to ALK inhibitors in patients with NSCLC reported that the proportion of patients with an MPR ≥ 80% ranged from 92 to 95% depending on the specific agent and whether the patients were naïve to ALK inhibitors (Ganti et al. 2022). Finally, among 21 patients with NSCLC who received oral chemotherapy in a prospective study, Jacobs et al. (2017) reported that 87% had good adherence (i.e., ≥ 90%), as measured with a medication event monitoring system. We are not aware of previous studies that reported adherence rates to osimertinib in this setting. We found lower rates of adherence as evaluated with the MGLS-4 than with other measures based on pill reconciliation or the PDC. Several studies in other therapeutic settings have shown a lack of correlation between medication adherence, as measured with questionnaires, and adherence, as measured by medication refill records (Cook et al. 2005; Gallagher et al. 2015; López-Simarro et al. 2016). In addition, we defined adherence with the MGLS-4 as patients who had not reported any problems with adherence (i.e., a score of 0 for all four items). Notably, the MGLS-4 has not been validated in patients with lung cancer, and its results could be highly influenced by the toxicities of oncologic therapies.

In the logistic regression models, the factors associated with treatment adherence were age, number of concomitant medications and time from treatment initiation of OAT. We found that for each year of increase in age, the likelihood of treatment adherence increased by 6%. In their systematic review of the literature on adherence to oral antineoplastic therapies, Greer et al. (2016) reported that either younger or older age was associated with poor adherence, although possibly the amount of evidence is greater against younger age. In a study conducted in the U.S. that evaluated treatment adherence and persistence with erlotinib in patients with NSCLC, Hess et al. (2017) reported no association between age and either adherence or persistence. In our analysis, the likelihood of adherence decreased by 16% for each additional concomitant medication. In their systematic review, Greer et al. (2016) reported that poor adherence is associated with oral antineoplastic treatment and either more concomitant medications or fewer concomitant medications. In the abovementioned study of erlotinib, the authors did not include concomitant medications in the multivariate models (Hess et al. 2017). The role of treatment duration in our study is difficult to evaluate because most patients were included while they were on treatment and not at treatment initiation. In our study, patients who were adherent had a longer time to disease progression during the first year of follow-up than did those who were nonadherent, and these results were supported by the multivariate analyses. The evidence regarding the impact of adherence on health outcomes in patients with cancer is limited and weak. Lasala and Santoleri (2022) systematically reviewed this topic and did not find any studies conducted in patients with NSCLC, with 28 of 42 studies performed in patients with hematological malignancies. Although the authors of this systematic review concluded that “a correlation between adherence and [clinical] outcomes has been widely demonstrated” (Lasala and Santoleri 2022), they did not provide a clear evaluation of the quality of the studies or the risk of publication bias. In contrast, considering the designs and heterogeneity of populations, medications, and measures of adherence used in these studies, we believe that the evidence supporting the potential association between adherence and better clinical outcomes in patients receiving OAT is weak.

Our study has several limitations. The sample size was calculated to estimate the prevalence of adherent patients, but it is too small to evaluate the factors associated with adherence, especially because of the low number of patients who were nonadherent. Most patients were included when they were already receiving OAT; thus, they were more likely to have a better prognosis. As research volunteers, patients were aware of being studied, which could have had an impact on their behavior; therefore, the Hawthorne effect could have increased the rate of adherence in our study, although the impact of this bias is difficult to estimate (McCambridge et al. 2014).

## Conclusion

Overall, consistent with previous evidence in this setting, our results seem to indicate that adherence to oral antineoplastic treatment among patients with NSCLC is high. Whether this high rate of adherence translates to better clinical outcomes, as suggested by our results regarding progression-free survival, should be further evaluated in larger samples.

## Electronic supplementary material

Below is the link to the electronic supplementary material.

Supplementary Material 1

## Data Availability

Data underlying the findings described in this manuscript may be obtained in accordance with AstraZeneca’s data sharing policy described at https://astrazenecagrouptrials.pharmacm.com/ST/Submission/Disclosure. Data for studies directly listed on Vivli can be requested through Vivli at www.vivli.org. Data for studies not listed on Vivli could be requested through Vivli at https://vivli.org/members/enquiries-about-studies-not-listed-on-the-vivli-platform/. AstraZeneca Vivli member page is also available outlining further details: https://vivli.org/ourmember/astrazeneca/.
