# Supplementary material for: Adherence to oral antineoplastic therapy among patients with advanced or metastatic non-small cell lung cancer: a noninterventional, prospective study
Source: J Cancer Res Clin Oncol. 2025 Aug 1;151(8):224. doi: 10.1007/s00432-025-06264-0 (PMC12316616; doi:10.1007/s00432-025-06264-0)

**Adherence to oral antineoplastic therapy among patients with advanced or metastatic non-small cell lung cancer: a noninterventional, prospective study**

Irene Mangues-Bafalluy, PhD^1^, Beatriz Bernardez Ferran, PharmD^2^, José Manuel Martínez Sesmero, PhD^3^, Andres Navarro-Ruiz, PhD^4^, Maria Teresa Martín-Conde, PhD^5^, Ana Rosa Rubio-Salvador, PhD^6^, Judith Rius-Perera, PharmD^1^, Marta Gilabert-Sotoca, PharmD^1^, Marta Domínguez López, MSc^7^, Angel Callejo Mellén, MSc^7^.

**Supplemental information**

**Supplementary Table 1**

**Multiple logistic regression analysis of the factors associated with treatment adherence >80%, including ‘time from treatment initiation of oral antineoplastic treatment’ (initial model)**

| **Variable** | **β** | **SE** | **Wald** | **df** | **p value** | **Odds ratio** | **95% CI** |
| --- | --- | --- | --- | --- | --- | --- | --- |
| Age | 0.058 | 0.023 | 6.700 | 1 | 0.010 | 1.060 | 1.014 to 1.108 |
| Sex (male)^a^ | -1.045 | 0.917 | 1.299 | 1 | 0.254 | .352 | 0.058 to 2.122 |
| Does any family member/caregiver/friend remind you daily or weekly to take your medication? (No)^a^ | 0.181 | 0.936 | 0.037 | 1 | 0.847 | 1.198 | 0.191 to 7.509 |
| EQ-5D-3L (not anxious or depressed)^a^ |  |  | 0.308 | 2 | 0.857 |  |  |
| EQ-5D-3L (I am moderately anxious or depressed) | -0.416 | 0.820 | 0.257 | 1 | 0.612 | .660 | 0.132 to 3.293 |
| EQ-5D-3L (I am extremely anxious or depressed) | 0.075 | 1.294 | 0.003 | 1 | 0.954 | 1.077 | 0.085 to 13.620 |
| How much control do you feel you have over your illness?^b^ | 0.023 | 0.132 | 0.030 | 1 | 0.862 | 1.023 | 0.790 to 1.326 |
| How much do you think your treatment can help your illness?^b^ | 0.283 | 0.271 | 1.085 | 1 | 0.298 | 1.327 | 0.779 to 2.258 |
| How well do you feel you understand your illness?^b^ | -0.295 | 0.210 | 1.988 | 1 | 0.159 | 0.744 | 0.494 to 1.122 |
| Number of concomitant medications per patient | -0.137 | 0.102 | 1.809 | 1 | 0.179 | 0.872 | 0.715 to 1.064 |
| Time since first oral antineoplastic treatment (months) | -0.028 | 0.017 | 2.528 | 1 | 0.112 | 0.973 | 0.940 to 1.006 |

^a^Reference categories appear in brackets

^b^Questions of the Brief Illness Perception Questionnaire, rated from 0 to 10; How much control do you feel you have over your illness? 0= absolutely no control and 10= extreme amount of control; How much do you think your treatment can help your illness? 0=not al all and 10=extremely helpful; How well do you feel you understand your illness? 0= don’t understand at all and 10= understand very clearly

CI, confidence interval; df, degrees of freedom; EQ-5D-3L, the 3-level version of the EQ-5D; SE, standard error

**Supplementary Table 2**

**Multiple logistic regression analysis of the factors associated with treatment adherence >80% without including ‘time from treatment initiation of oral antineoplastic treatment’ (initial model)**

| **Variable** | **β** | **SE** | **Wald** | **df** | **p value** | **Odds**  **ratio** | **95% CI** |
| --- | --- | --- | --- | --- | --- | --- | --- |
| Age | 0.049 | 0.019 | 6.732 | 1 | 0.009 | 1.051 | 1.012 to 1.090 |
| Sex (male)^a^ | -0.949 | 0.776 | 1.496 | 1 | 0.221 | 0.387 | 0.085 to 1.771 |
| Does any family member/caregiver/friend remind you daily or weekly to take your medication? (no)^a^ | 0.710 | 0.859 | 0.684 | 1 | 0.408 | 2.034 | 0.378 to 10.947 |
| EQ-5D-3L (not anxious or depressed)^a^ |  |  | 0.192 | 2 | 0.908 |  |  |
| EQ-5D-3L (I am moderately anxious or depressed) | -0.197 | 0.689 | 0.082 | 1 | 0.774 | 0.821 | 0.213 to 3.166 |
| EQ-5D-3L (I am extremely anxious or depressed) | 0.293 | 1.244 | 0.055 | 1 | 0.814 | 1.340 | 0.117 to 15.357 |
| How much control do you feel you have over your illness?^b^ | 0.007 | 0.116 | 0.004 | 1 | 0.950 | 1.007 | 0.802 to 1.265 |
| How much do you think your treatment can help your illness?^b^ | 0.122 | 0.190 | 0.410 | 1 | 0.522 | 1.129 | 0.778 to 1.638 |
| How well do you feel you understand your illness?^b^ | -0.129 | 0.131 | 0.972 | 1 | 0.324 | 0.879 | 0.681 to 1.136 |
| Number of concomitant medications per patient | -0.174 | 0.088 | 3.860 | 1 | 0.049 | 0.841 | 0.707 to 1.000 |

^a^Reference categories appear in brackets

^b^Questions of the Brief Illness Perception Questionnaire, rated from 0 to 10; How much control do you feel you have over your illness? 0= absolutely no control and 10= extreme amount of control; How much do you think your treatment can help your illness? 0=not al all and 10=extremely helpful; How well do you feel you understand your illness? 0= don’t understand at all and 10= understand very clearly

CI, confidence interval; df, degrees of freedom; EQ-5D-3L, the 3-level version ofthe EQ-5D; SE, standard error

**Supplementary Figure 1**

**Time to progression in adherent and nonadherent patients to oral antineoplastic treatment for non-small cell lung cancer from treatment initiation**


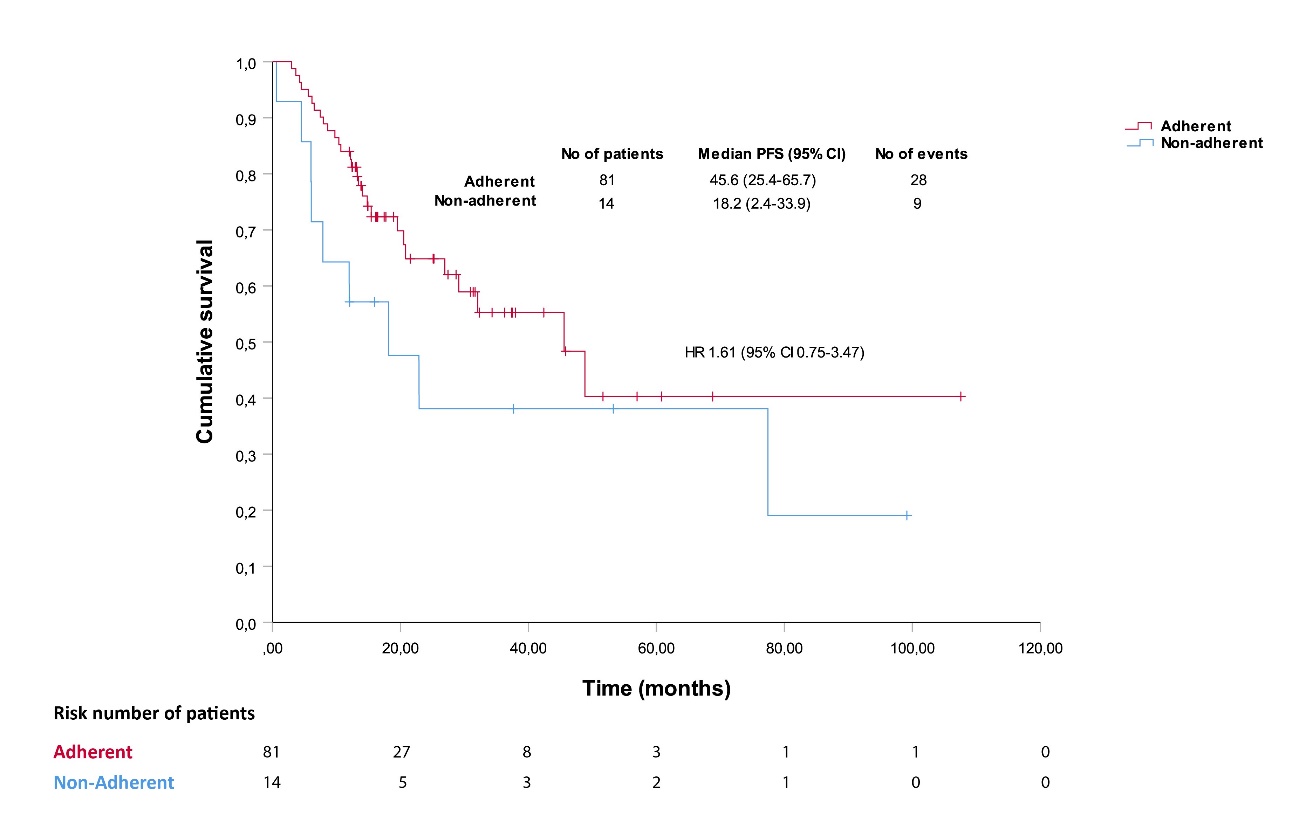

Supplement: Supplementary file 1 — Supplementary Material 1 [file 432_2025_6264_MOESM1_ESM.docx]
